# Shift in competitive ability mediated by soil biota in an invasive plant

**DOI:** 10.1002/ece3.8287

**Published:** 2021-11-02

**Authors:** Fangfang Huang, Qiaoqiao Huang, Xianhua Gan, Weiqiang Zhang, Yuedong Guo, Yuhui Huang

**Affiliations:** ^1^ Guangdong Provincial Key Laboratory of Silviculture, Protection and Utilization Guangdong Academy of Forestry Guangzhou China; ^2^ Key Laboratory of Integrated Pest Management on Tropical Crops Ministry of Agriculture and Rural Affairs Environment and Plant Protection Institute Chinese Academy of Tropical Agricultural Sciences Haikou China

**Keywords:** competition environment, competitive ability, *Mikania micrantha*, plant invasion, soil biota

## Abstract

Understanding the shifts in competitive ability and its driving forces is key to predict the future of plant invasion. Changes in the competition environment and soil biota are two selective forces that impose remarkable influences on competitive ability. By far, evidence of the interactive effects of competition environment and soil biota on competitive ability of invasive species is rare. Here, we investigated their interactive effects using an invasive perennial vine, *Mikania micrantha*. The competitive performance of seven *M*. *micrantha* populations varying in their conspecific and heterospecific abundance were monitored in a greenhouse experiment, by manipulating soil biota (live and sterilized) and competition conditions (competition‐free, intraspecific, and interspecific competition). Our results showed that with increasing conspecific abundance and decreasing heterospecific abundance, (1) *M*. *micrantha* increased intraspecific competition tolerance and intra‐ vs. interspecific competitive ability but decreased interspecific competition tolerance; (2) *M*. *micrantha* increased tolerance of the negative soil biota effect; and (3) interspecific competition tolerance of *M*. *micrantha* was increasingly suppressed by the presence of soil biota, but intraspecific competition tolerance was less affected. These results highlight the importance of the soil biota effect on the evolution of competitive ability during the invasion process. To better control *M*. *micrantha* invasion, our results imply that introduction of competition‐tolerant native plants that align with conservation priorities may be effective where *M*. *micrantha* populations are long‐established and inferior in inter‐ vs. intraspecific competitive ability, whereas eradication may be effective where populations are newly invaded and fast‐growing.

## Graphical abstract

This work investigated the interactive effects of competitive environment and soil biota on competitive ability in an invasive plant *Mikania micrantha*. The results showed that *M*. *micrantha* evolved higher intra‐ vs. interspecific competitive ability during its invasion, and that soil biota likely mediated the evolutionary process. This work implies that introduction of competition‐tolerant native plants that align with conservation priorities into long established invaded sites may be effective to control the invasion of *M*. *micrantha*.

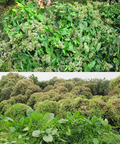

## 1 INTRODUCTION

Explaining the success of introduced species has long been a focus in invasion ecology. Many theories on this topic posit that the evolutionary mismatch between invasive species and native communities releases invasive species from ecological factors that limit invasive species in native ranges (e.g., competition, pathogens, and herbivores; Hallett, 2006; Maron & Vilà, 2001). Changes in selective pressures in new ranges allow invasive species to evolve to be more competitive by investing more resource into growth and reproduction than defense (Blossey & Nötzold, 1995; Montesinos et al., 2019; Qin et al., 2013; te Beest et al., 2009), contributing to the early success of invasive species. However, evolutionary shifts in competition‐related traits can take place not only between invaded and native ranges but also within invaded ranges over time. For example, invasive populations of *Alliaria petiolata* tended to evolve lower concentrations of allelochemicals over time across over 50‐year invasion (Lankau et al., 2009); older populations of invasive *Impatiens glandulifera* within the invasive range produced greater secondary defense compound than more recently established populations (Gruntman et al., 2017). Considering the importance of competitive ability to invasion success in invaded ranges, understanding the shifts in competitive ability and its driving forces is key to understand the future of plant invasion.

Invasive plants often experience a rapid shift in the relative abundance between invasive populations vs. co‐occurring plants, imposing strong selective pressures on the evolution of inter‐ vs. intraspecific competitive ability (Lankau & Strauss, 2011). Commonly, at sites where invasive species encounter higher hetero‐ vs. conspecific neighbors (usually recently invaded sites and invasion edges), traits conferring high inter‐ vs. intraspecific competitive ability (e.g., allelopathy and defense against generalist enemies) may be favored; on the contrary, at sites of intense monospecific stands of invasive species (usually long invaded sites and invasion center), selections may favor traits conferring high intra‐ vs. interspecific competitive ability (e.g., reduced allelopathy and defense against specialist enemies; Lankau et al., 2009; Wan et al., 2019). As the direction and magnitude of selective pressures by intra‐ and interspecific competitions vary across different spatiotemporal invasion contexts, the competitive ability is likely to evolve differentially among invasive populations (Burton et al., 2010).

In addition, soil biota play an important role in the competitive performance of invasive species (Dawson & Schrama, 2016; Hilbig & Allen, 2015; Perkins & Nowak, 2012). When first invaded to new ranges, invasive species may experience a less negative or even positive soil biota effect by establishing novel interactions with local soil biota (e.g., release from soil enemies from native ranges and enhanced mutualistic dependence; Eppinga et al., 2006; Inderjit & van der Putten, 2010; Reinhart & Callaway, 2006), which benefit its competitive ability. This advantage, however, may attenuate over time as high loads of soil enemies accumulate with dense patches of invasive species (Hawkes, 2007; Lau & Suwa, 2016; Mitchell et al., 2010), and/or mutualistic associations become less beneficial (Luo et al., 2021; Waller et al., 2016). For example, an invasive plant *Heracleum mantegazzianum* had lower survival, biomass, and competitive ability when it was grown in soil inocula from earlier invaded sites (Dostál et al., 2013). The increasingly negative soil biota effect may therefore incur resource shifting from growth back to defense in invasive species and ultimately influence competitive ability.

By far, evidence on the interactive effects of populations’ competition environment and soil biota affecting competitive ability of invasive species is rare, although previous studies have indicated that the soil biota effect depends on competitor's identity (Bezemer et al., 2018; Lekberg et al., 2018). Linking with the invasion process, we predict that in newly invaded areas with mostly heterospecific neighbors, invasive species may preferentially interact with soil microbial components that are beneficial to enhance interspecific competitive ability and/or suppress components that reduce interspecific competitive ability, but resources allocated to soil biota interactions should be at a relatively low level as the soil biota effect is supposed to be less antagonistic or even beneficial; in long invaded areas with mostly conspecific competitors, invasive species may tend to be increasingly tolerant of the negative soil biota effect to maintain intraspecific more than interspecific competitive ability.

**FIGURE 1:**
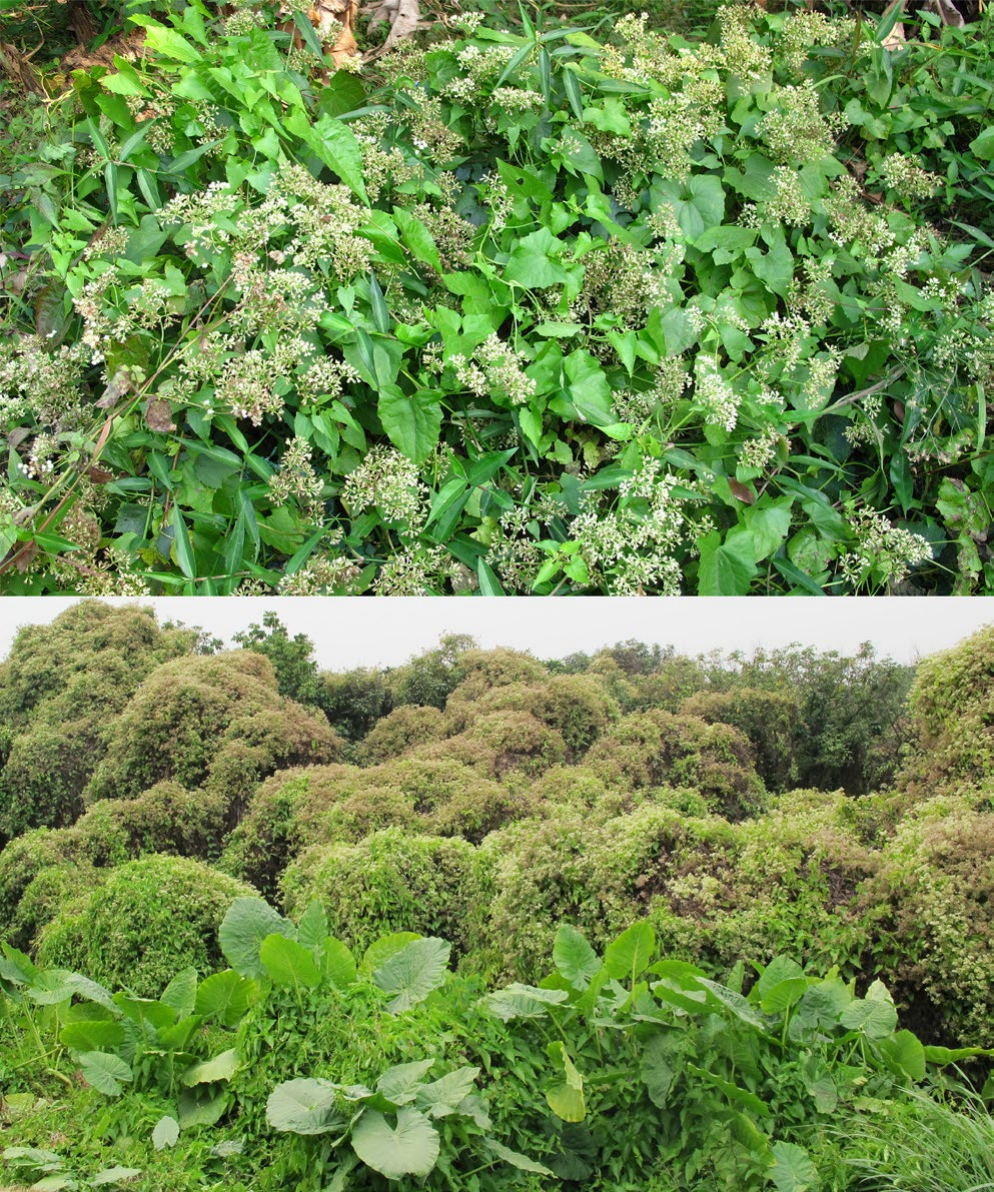
*Mikania micrantha*, a highly invasive vine in South China. Photo credit Fangfang Huang

In this study, we investigated the interactive effects of competition environment and soil biota on competitive ability using an invasive perennial vine, *Mikania micrantha* (Figure 1). Commonly identified as one of the worst weeds in the world, *M*. *micrantha* originated from tropical Central and South America and has invaded throughout much of the tropics (Lowe et al., 2000). The vine was intentionally introduced to Hong Kong in the late 19th century, and it began to spread in the 1950s–1960s (Kong et al., 2000). *M*. *micrantha* has now widely spread throughout the coastal region of Guangdong Province (Wang et al., 2003; Zhang et al., 2004). The competitive advantage of *M*. *micrantha* in introduced ranges is partially due to allelopathic effects, which significantly alter the dynamics of underground soil biota communities and inhibit the growth of native competitors (Chen et al., 2013; Li et al., 2006; Shao et al., 2005). Recent studies found that the intraspecific competitive ability of *M*. *micrantha* shifted in response to the changes in conspecific competition intensity during its invasion in South China (Huang & Peng, 2016).

**FIGURE 2:**
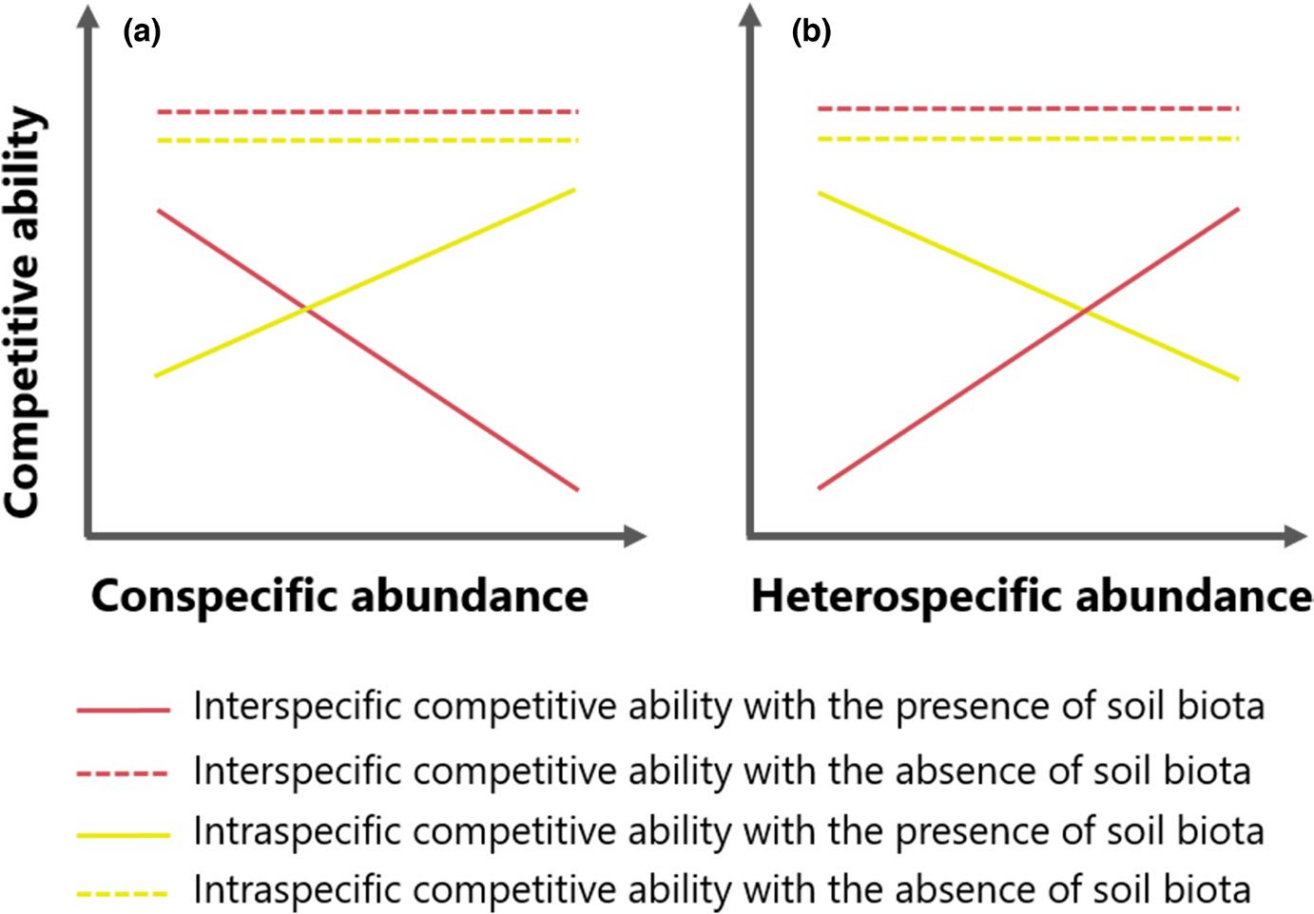
Diagram of hypothesis on shifts in competitive ability along conspecific and heterospecific abundance. In this study, biomass was used as the proxy of competitive ability. We hypothesized that soil biota mediated the evolutionary shifting in intra‐ and interspecific competitive ability. In areas with high‐heterospecific and low‐conspecific abundance (usually newly invaded sites; toward left end in (a) and right end in (b)), invasive species may tend to preferentially interact with soil components that are beneficial to enhance interspecific competitive ability and/or suppress soil components that reduce interspecific competitive ability, although resources allocated to soil biota interactions should be at a relatively low level as the soil biota effect is supposed to be less antagonistic or even beneficial; by contrast, in areas with low‐heterospecific and high‐conspecific abundance (usually long invaded sites; toward right end in (a) and left end in (b)), invasive species may tend to be more tolerant of the negative soil effect to maintain intraspecific more than interspecific competitive ability. Accordingly, interspecific competitive ability may be increasingly suppressed by the presence of soil biota as the competition environment shifting from heterospecific to conspecific dominant; on the contrary, intraspecific competitive ability may be less suppressed during this process

We grew seven *M*. *micrantha* populations varying in their field conspecific and heterospecific abundance by manipulating soil biota (live and sterilized) and competition conditions (competition‐free, intra‐, and interspecific competition) in a greenhouse experiment. We asked the following questions: (1) How is *M*. *micrantha* competitive performance related to its conspecific and heterospecific abundance? (2) What is the effect of soil biota on *M*. *micrantha* competitive performance? Does the effect depend on competition environment? We hypothesized that with increasing conspecific abundance and decreasing heterospecific abundance, (1) *M*. *micrantha* increased intraspecific competitive ability but decreased interspecific competitive ability; (2) the effect of soil biota was competition environment‐dependent; specifically, interspecific competitive ability was increasingly more suppressed by the presence of soil biota relative to intraspecific competitive ability (see Figure 2 for diagram of hypothesis).

## 2 MATERIAL AND METHODS

### 2.1 Plant and soil sources

Seven *M*. *micrantha* populations covering a wide range of percent covers of *M*. *micrantha* and heterospecific plants were selected as focal populations in the greenhouse experiment (see Table S1 for population information) based on a field survey from December 2016 to February 2017 with available distribution data (Huang et al., 2015; Wang et al., 2003). For each of the seven populations, seeds were collected from multiple inflorescences, and the percent covers of *M*. *micrantha* and heterospecific plant species were estimated from 5 to 8.1 m^2^ plots. Percent cover was used to represent the competition environment (Huang et al., 2015; Lankau, 2012). It must be admitted that percent cover may not provide an exact measure as the competition environment is dynamic for *M*. *micrantha*. However, over a wide span of conspecific and heterospecific abundance, percent cover can serve as a useful predictor of the relative competition environment for comparative studies.

To set up intra‐ vs. interspecific competition treatments, the seeds of an invasive annual *Bidens pilosa*, a native perennial *Polygonum chinensis*, and a competing *M*. *micrantha* population were collected from an additional site, which was over 10 km away from the seven focal populations. *B*. *pilosa* and *P*. *chinensis* were selected as heterospecific competitors because they commonly coexist with *M*. *micrantha* in the field and are common in forests and abandoned areas in South China. The levels of conspecific and heterospecific abundance at this site were moderate, so the potential competition bias for the seven focal populations was minimized. All seeds were air‐dried and stored at room temperature until the greenhouse experiment began.

The soil material was collected from three sites of different invasion histories in May 2017 (see Table S2 for soil source information). In each site, bulk soil was collected (c. 20 L at 0–20 cm depth) from four locations at least 30 m apart. Matter such as plants, rocks, and earthworms were removed from the soil. Soil collections within each site were homogenized, sieved, and stored at 4°C fewer than 10 days before the experiment began. To set up live vs. sterilized soil treatments, half of the soil samples from each site were autoclaved twice at 121°C for 60 min with a 24‐h rest period in between. Both live and sterilized soil samples were analyzed for 10 soil chemical variables: pH, organic matter, ammonium and nitrate nitrogen, available P, available K, exchangeable Ca, exchangeable Mg, available Zn, and available Mn (see Table S3 for soil chemical information).

### 2.2 Experimental design

Seeds of *M*. *micrantha*, *B*. *pilosa*, and *P*. *chinensis* were germinated in trays with sterilized potting soil in early May 2017. Seeds from different populations did not germinate simultaneously, and there was about one‐week gap in germination time between the earliest and latest populations. To minimize the effect of seedling size on competition result due to difference in germination time, seedlings of similar size were selected in the experiment, and initial size of each plant (ranking 1–5 from the smallest to largest) was recorded when it was transplanted after three weeks of growth. Each pot was filled with 550 ml of sterilized potting soil, inoculated with 70 ml of field‐collecting live soil or an autoclaved sample of the same soil, and topped with 80 ml of sterilized potting soil. In both live and sterilized soil, *M*. *micrantha* plants were grown either alone or with intra‐ or interspecific competitors. To simulate intraspecific competition, each *M*. *micrantha* plant from the seven focal populations was grown with two competing *M*. *micrantha* plants from the additional site. To simulate interspecific competition, each *M*. *micrantha* plant was grown with a *B*. *pilosa* and a *P*. *chinensis* plant. Each competition × soil treatment combination was repeated for the three aforementioned soil sources. Such was done to minimize potential soil sample bias as soil biota can vary from site to site (Maron et al., 2015). Five replicates were set for each treatment, yielding 630 pots in total (7 *M. micrantha* populations × 3 competition treatments × 2 soil treatments × 3 soil sources × 5 replicates = 630 pots; see Figure S1 for experimental design). A 1‐m stick was set in each pot to support *M*. *micrantha* climbing. Seedlings were replaced if they died in the first week after transplanting. Pots were watered once daily. Within each of the five blocks, the plants were randomly arranged and repositioned every week. The pots were grown for three months, after which the above‐ and belowground biomass (mainly tap roots as fine roots were tightly intertwined and difficult to separate) of both focal and neighbor plants were harvested. The biomass was dried at 60°C for 72 h and weighed. Fourteen plants died during the experiment and their pots were removed.

Plant biomass was used as the proxy of competitive ability. Fecundity‐related traits such as seed production and flowering phenology, which were often used to measure competitive ability (e.g., Alexander & Levine, 2019), were not measured in this study. As *M*. *micrantha* is highly invasive, we harvested all plants before *M*. *micrantha* flowered to eliminate unintentional spread.

As the same soil inoculum was used in our experiment, the difference in response to soil biota reflected the genetic difference among *M*. *micrantha* populations, rather than the difference in the pool of soil biota. It should be noted that we did not intend to test local adaptation to soil biota, but rather to test the difference in the relationship with soil biota among populations. Therefore, we used the same three soil sources, instead of using local soil of each population.

### 2.3 Data analysis

The autoclave procedure may increase soil nutrient for sterilized soil, although the amount of soil inoculum was small. To account for the potential effect that the difference in soil chemistry may have on competitive performance due to the autoclave procedure, PCA was performed to reduce the 10 soil chemical variables to one principal component that retained over 90% of the original variation. Soil PC1 was included in the following analysis. In this way, differences between live and sterilized soils among populations should reflect the effect of soil biota, rather than the differences in soil chemistry caused by the autoclave.

To test how the growth of *M*. *micrantha* varied in response to the competition environment and soil biota, a linear mixed‐effects model was run with *M*. *micrantha* biomass as the response variable; average percent cover of *M*. *micrantha* population, competition treatment, soil treatment and their two‐ and three‐way interactions, and soil source as the fixed factors; initial size of focal *M*. *micrantha* plant and soil PC1 as the covariates; and *M*. *micrantha* population and block as the random factors.

Two indicators were adopted to evaluate the variation in competitive ability. First, the indicator of relative interaction intensity (RII; Armas et al., 2004) was used to estimate competition tolerance by comparing biomass in the presence vs. absence of competition. RII was calculated for each focal *M*. *micrantha* population under different competition and soil treatments as follows:

$$\text{Relative}\;\text{interaction}\;\text{intensity}=\frac{B_{C}‐B_{N}}{B_{C}+B_{N}}$$

where B_C_ and B_N_ are the biomass of the focal *M*. *micrantha* plant in the presence and absence of competition, respectively. A more negative value indicates higher levels of competition intolerance in focal plants. A linear mixed‐effects model was run with RII as the response variable; *M*. *micrantha* percent cover, competition treatment, soil treatment and their two‐ and three‐way interactions. and soil source as the fixed factors; initial size of the focal *M*. *micrantha* plant in the presence and absence of competition and soil PC1 as the covariates; and *M*. *micrantha* population and block as the random factors.

Second, the indicator of relative competitive ability (RCA) was used to estimate intra‐ vs. interspecific competitive ability. RCA was calculated for each focal population in live and sterilized soils as follows:

$$\text{Relative}\;\text{competitive}\;\text{ability}=\frac{\text{biomass}\;\text{in}\;\text{intraspecific}\;\text{competition}}{\text{biomass}\;\text{in}\;\text{interspecific}\;\text{competition}}$$

A value of 1 indicates equivalent intra‐ vs. interspecific competitive ability. A linear model was run with *M*. *micrantha* relative competitive ability was the response variable, *M*. *micrantha* percent cover, soil treatment and their two‐way interactions, and soil source as the fixed factors; initial size of focal *M*. *micrantha* plants in intra‐ and interspecific competition and soil PC1 as the covariates; and population and block as the random factors.

The aforementioned models were rerun but substituted the heterospecific plants percent cover in place of the *M*. *micrantha* percent cover to fully test the effect of the competition environment on competitive ability. Last, because correlated environmental factors may confound the interpretation of the results, to explore this possibility, the aforementioned models were rerun with longitude, latitude, and altitude of *M*. *micrantha* population as the additional covariates. All analyses were performed in JMP 16 Pro (SAS institute, Cary, NC, USA).

## 3 RESULTS

### 3.1 Effect of competition environment on *M*. *micrantha* competitive ability

**FIGURE 3:**
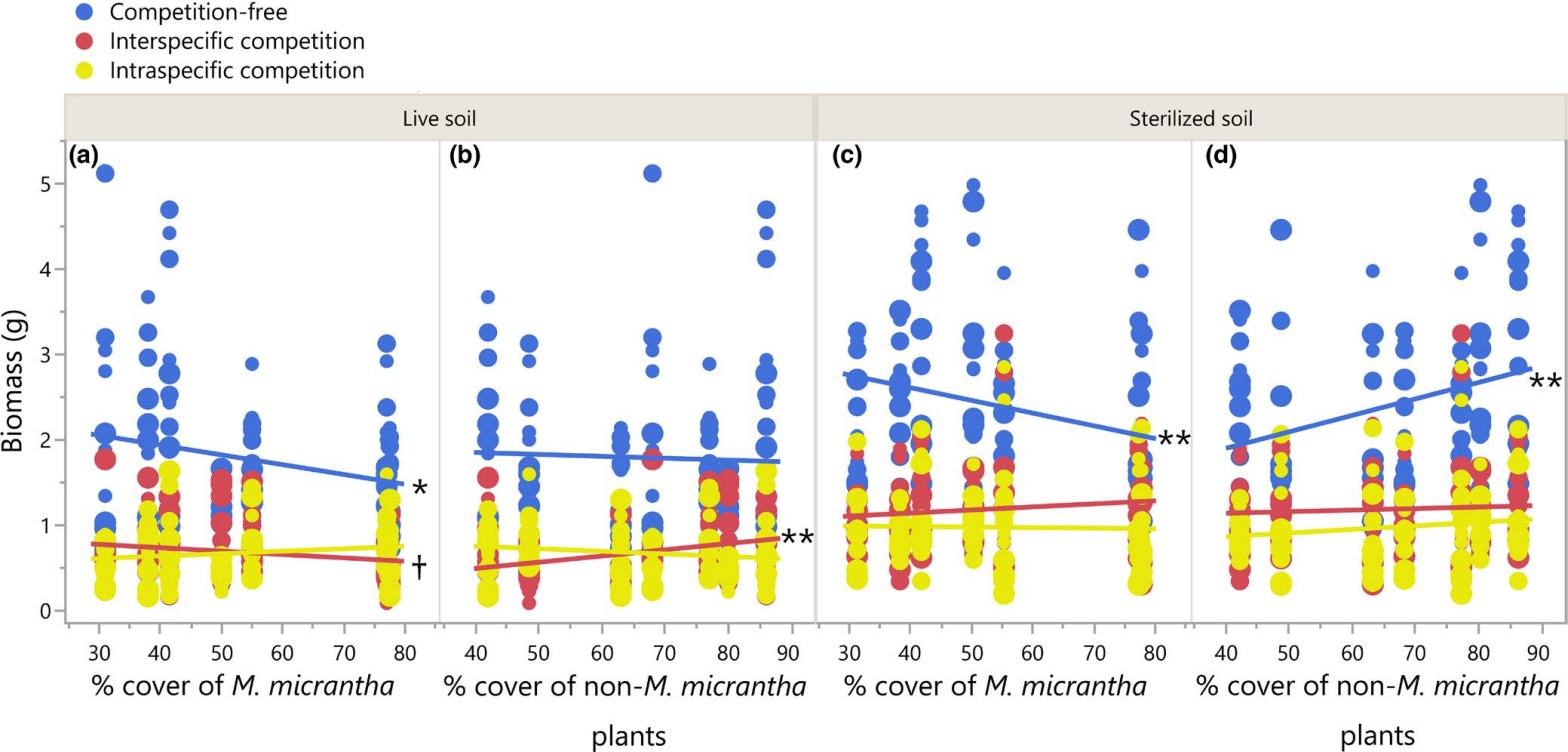
*Mikania micrantha* biomass along conspecific and heterospecific percent covers at the source sites under different competition and soil treatments. (a) and (b), respectively, show the biomass along conspecific and heterospecific percent covers in live soil; (c) and (d) show the biomass along conspecific and heterospecific percent covers in sterilized soil. Symbol size indicates invasion time of the three soil‐collecting sites. Larger size denotes longer estimated invasion time (see Table S2 for estimated invasion time for each site). Significant and marginally significant regressions were denoted at the right end of the regression lines: **p ≤ .01, *.01 < *p* ≤ .05, ^†^.05 < *p* ≤ .10

*Mikania micrantha* populations from high conspecific abundance sites tended to produce higher biomass in intraspecific competition but lower biomass in interspecific competition environments, compared with those populations from low conspecific abundance sites in live soil (significant conspecific percent cover × competition effect in Table 1, Figure 3a); Likewise, *M*. *micrantha* populations from high heterospecific abundance sites tended to produce higher biomass in interspecific competition but lower biomass in intraspecific competition compared with the populations from the other end (marginally significant heterospecific percent cover × competition effect in Table 1, Figure 3b).

**TABLE 1:** Linear mixed‐effects model result of the effect of *Mikania micrantha*/non‐*M*. *micrantha* percent covers, competition, and soil treatments on *M*. *micrantha* biomass

Effect	*df*	*F*	*p*
*M. micrantha* % cover (conspecific cover)	1	3.286	.129
Competition treatment (competition)	2	301.928	<.001
Soil treatment (soil)	1	66.335	<.001
Conspecific cover × competition	2	12.351	<.001
Conspecific cover × soil	1	0.222	.638
Competition × soil	2	5.614	.004
Conspecific cover × competition × soil	2	1.782	.169
Initial size of focal *M*. *micrantha* plant	1	10.156	.002
Soil source	2	2.558	.078
Soil PC1	1	0.278	.599
			
Non‐*M*. *micrantha* plant % cover (heterospecific cover)	1	3.468	.121
Competition treatment (competition)	2	299.908	<.001
Soil treatment (soil)	1	65.298	<.001
Heterospecific cover × competition	2	2.856	.058
Heterospecific cover × soil	1	5.286	.022
Competition × soil	2	5.468	.004
Heterospecific cover × competition × soil	2	6.626	.001
Initial size of focal *M*. *micrantha* plant	1	9.969	.002
Soil source	2	2.455	.087
Soil PC1	1	0.257	.613

**FIGURE 4:**
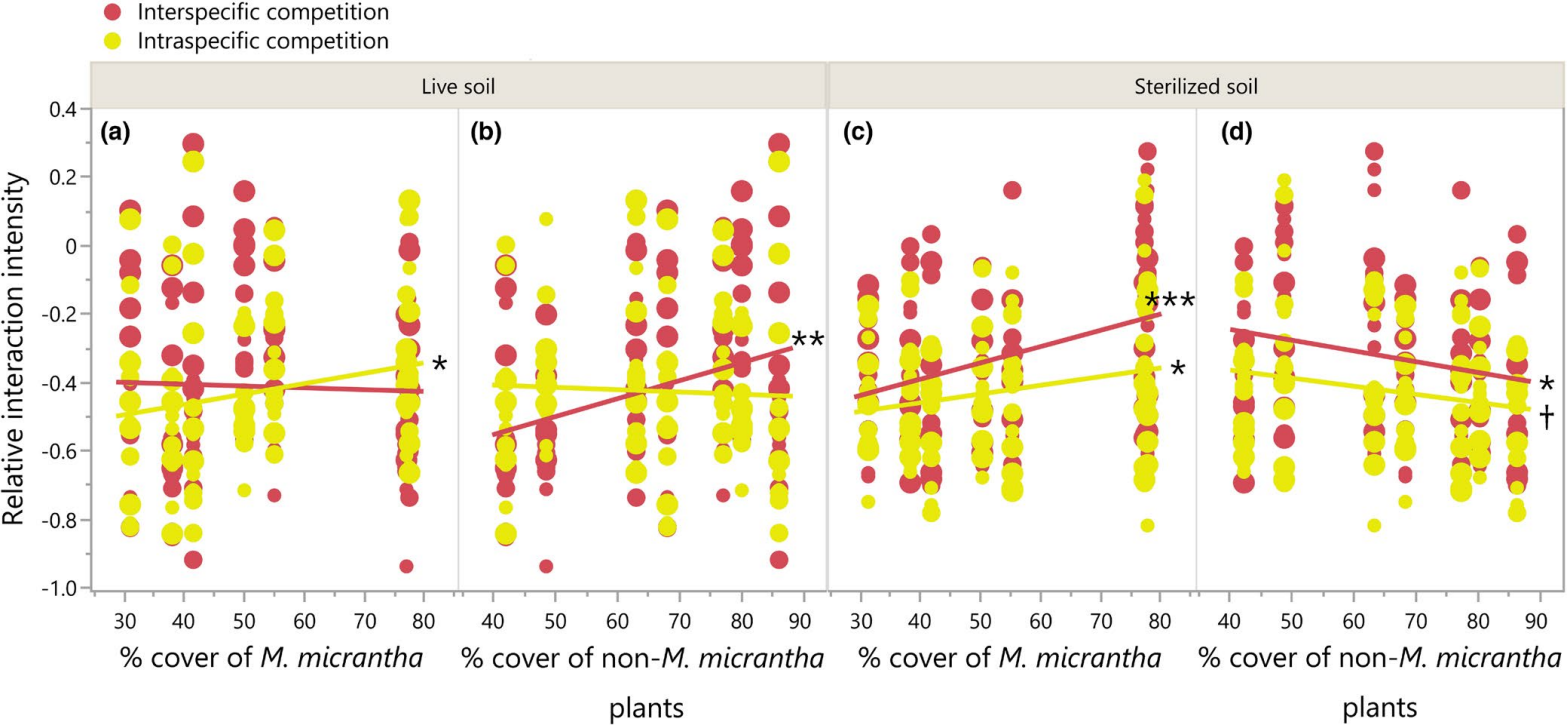
*Mikania micrantha* relative interaction intensity (RII) along conspecific and heterospecific percent covers under different competition and soil environments. (a) and (b), respectively, show the RIIs along conspecific and heterospecific percent covers in live soil; (c) and (d) show the RIIs along conspecific and heterospecific percent covers in sterilized soil. **p ≤ .01, *.01 < *p* ≤ .05, ^†^.05 < *p* ≤ .10

**FIGURE 5:**
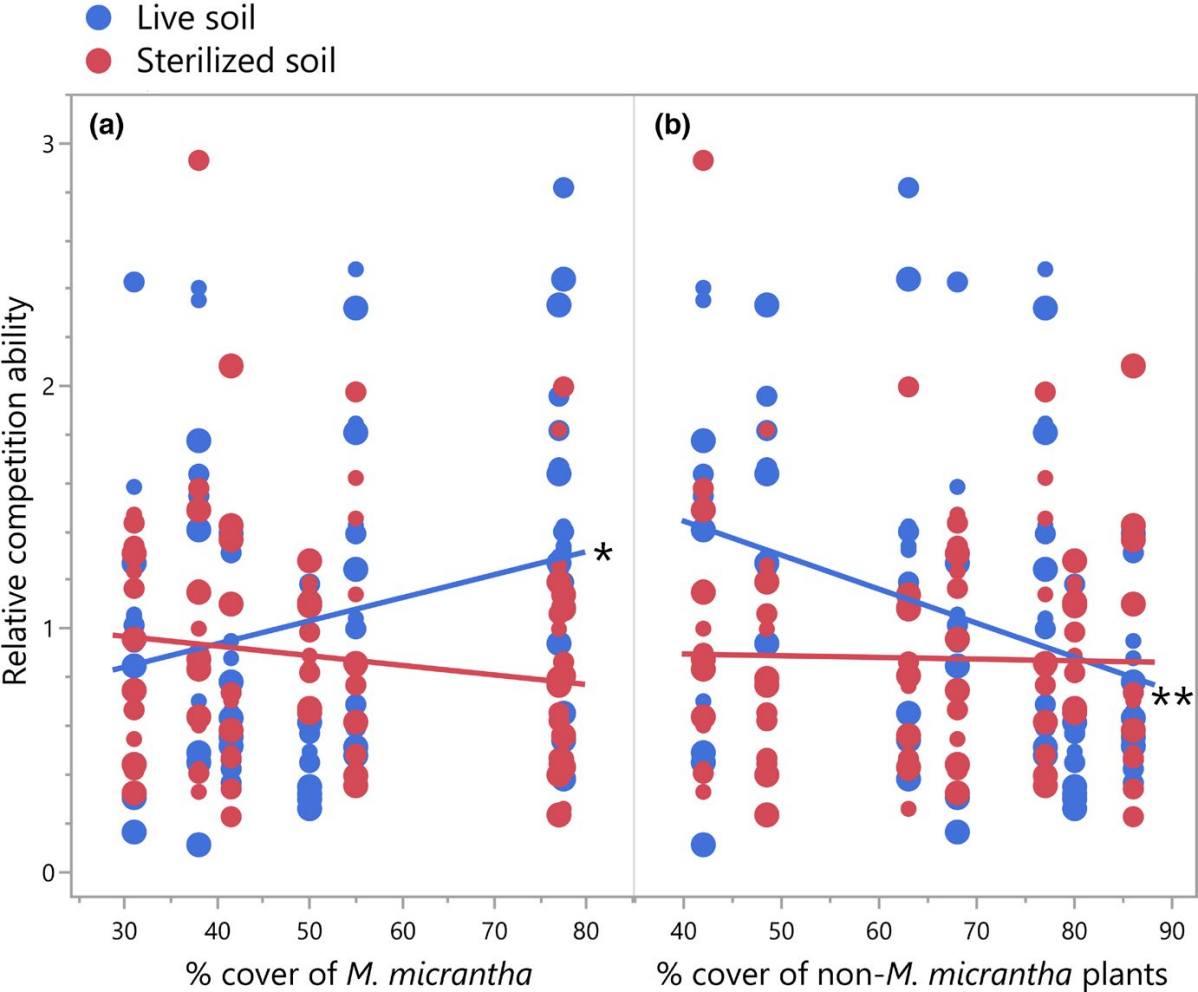
*Mikania micrantha* relative competitive ability along conspecific and heterospecific percent covers in live and sterilized soils. (a) relative competitive ability along conspecific percent cover; (b) relative competitive ability along heterospecific cover. **p ≤ .01, *.01 < *p* ≤ .05

Consistent with the biomass results, *M*. *micrantha* populations from high conspecific abundance sites had higher intraspecific competition tolerance in live soil (Table 2, Figure 4a), compared with populations from low conspecific abundance sites; populations from high‐heterospecific abundance sites had higher interspecific competition tolerance than populations from the other end (Figure 4b). Although running in the same directions, these patterns may derive differently: the increased intraspecific competition tolerance was more related to B_N_ (i.e., decreased biomass along conspecific cover in competition‐free environment, Figure 3a), whereas the increased interspecific competition tolerance was more related to B_C_ (Figure 3b). *M*. *micrantha* populations from high‐conspecific abundance and low‐heterospecific abundance sites tended to have higher intra‐ vs. interspecific competitive ability with the presence of soil biota than those from low‐conspecific abundance and high‐heterospecific abundance sites (Table 3, Figure 5).

**TABLE 2:** Linear mixed‐effects model result of the effect of *Mikania micrantha*/non‐*M*. *micrantha* percent covers and soil treatment on relative interaction intensity

Effects	*df*	*F*	*p*
*M. micrantha* % cover (conspecific cover)	1	231.696	<.001
Competition treatment (competition)	1	6.754	.010
Soil treatment (soil)	1	24.613	<.001
Conspecific cover × competition	1	0.263	.608
Conspecific cover × soil	1	2.595	.108
Competition × soil	1	3.172	.076
Conspecific cover × competition × soil	1	4.727	.030
Soil source	2	18.000	<.001
Soil PC1	1	36.116	<.001
Initial size of focal *M*. *micrantha* in the absence of competition	1	34.800	<.001
Initial size of focal *M*. *micrantha* in the presence of competition	1	5.981	.017
			
Non‐*M*. *micrantha* plant % cover (heterospecific cover)	1	0.007	.936
Competition treatment (competition)	1	7.748	.006
Soil treatment (soil)	1	20.688	<.001
Heterospecific cover × competition	1	2.431	.120
Heterospecific cover × soil	1	12.034	<.001
Competition × soil	1	3.470	.063
Heterospecific cover × competition × soil	1	4.763	.030
Soil source	2	18.876	<.001
Soil PC1	1	31.413	<.001
Initial size of focal *M*. *micrantha* in the absence of competition	1	4.874	.028
Initial size of focal *M*. *micrantha* in the presence of competition	1	8.029	.005

**TABLE 3:** Linear mixed‐effects model result of the effect of *M*. *micrantha*/non‐*M*. *micrantha* percent covers and soil treatment on relative competitive ability

Effect	*df*	*F*	*p*
*M. micrantha* % cover (conspecific cover)	1	0.290	.613
Soil treatment (soil)	1	11.661	<.001
Conspecific cover ×soil	1	6.411	.012
Initial size of *M*. *micrantha* plant in interspecific competition	1	0.776	.380
Initial size of *M*. *micrantha* plant in intraspecific competition	1	3.644	.058
Soil source	2	1.912	.151
Soil PC1	1	3.876	.051
			
Non‐*M*. *micrantha* plant % cover (heterospecific cover)	1	13.837	.015
Soil treatment (soil)	1	11.215	.001
Heterospecific cover × soil	1	9.312	.003
Initial size of *M*. *micrantha* plant in interspecific competition	1	2.058	.156
Initial size of *M*. *micrantha* plant in intraspecific competition	1	1.750	.189
Soil source	2	1.640	.197
Soil PC1	1	3.248	.073

### 3.2 Effect of soil biota on *M*. *micrantha* growth

*Mikania micrantha* biomass was significantly reduced by the presence of soil biota in all competition environments to different degrees (all *p* < .01; 27% biomass reduction in live vs. sterilized soil in competition‐free environment, 42% in interspecific competition and 31% in intraspecific competition; Table 1, Figure S2), suggesting the overall negative soil biota effect on *M*. *micrantha* growth.

### 3.3 Interactive effects of competition environment and soil biota on *M*. *micrantha* competitive ability

Significant interactive effects of the competition environment and soil biota on *M*. *micrantha* competitive ability were observed. For the biomass results, the three‐way interactive effect of non‐*M*. *micrantha* plants percent cover, competition, and soil treatment was significant (Table 1), suggesting that the extent to how *M*. *micrantha* biomass was reduced by soil biota differed among populations from sites varied in heterospecific abundance. Specifically, biomass of *M*. *micrantha* populations from high‐heterospecific abundance sites was less reduced by the presence of soil biota when competing interspecifically but was greater reduced when growing alone, compared with those from low‐heterospecific abundance sites (Figure 3b,d). Reverse trends were detected with increasing *M*. *micrantha* percent cover (Figure 3a,c), that is, biomass of populations from sites of high conspecific abundance was less reduced by soil biota when growing alone but was more reduced when competing interspecifically than populations from the other end (although the interaction effect of *M*. *micrantha* percent cover, competition, and soil treatment was insignificant; Table 1). In addition, regressions indicated that the trends in biomass along conspecific and heterospecific abundance in intraspecific competition were not significant in either live or sterilized soil (Figure 3).

*Mikania micrantha* relative interaction intensity was significantly affected by competition treatment, soil treatment, and population's competition environment (Table 2). The presence of soil biota reduced interspecific competition tolerance more for populations from high conspecific and low heterospecific abundance sites than for populations from the other ends; however, it did not alter the trends in intraspecific competition tolerance (Figure 4). The patterns in sterilized soil were related more to B_N_ than to B_C_ as there was no difference in biomass detected among *M*. *micrantha* populations when they were in competition (Figure 3c,d).

Last, *M*. *micrantha* relative competitive ability was significantly influenced by soil treatment and competition environment (Table 3). Trends in relative competitive ability along with conspecific/heterospecific abundance in live soil disappeared when soil biota was removed (Figure 5). In addition, populations from high‐conspecific and low‐heterospecific abundance sites suffered greater reductions in relative competitive ability with removal of soil biota than populations from the other ends (Figure 5), likely due to a greater bounce in biomass in the inter‐ vs. intraspecific competition environment when soil biota was removed (Figure 3). The inclusion of geographic factors into models did not quantitatively change the results (Tables S4–S6), suggesting that the observed patterns did not result from confounding environmental factors.

## 4 DISCUSSION

In the present study, we investigated the interactive effects of the competition environment and soil biota on *M*. *micrantha* competitive ability. Consistent with our hypotheses, the results showed that with increasing conspecific abundance and decreasing heterospecific abundance, (1) *M*. *micrantha* increased intraspecific competition tolerance and intra‐ vs. interspecific competitive ability but decreased interspecific competition tolerance; (2) *M*. *micrantha* increased tolerance of the negative soil biota effect, evidenced by the growth being less inhibited by the presence of soil biota when growing in isolation; (3) interspecific competition tolerance was increasingly suppressed by the presence of soil biota, but intraspecific competition tolerance was less evidently affected. Together, our results strongly suggest that soil biota mediate the evolutionary shifts in intra‐ and interspecific competitive ability of *M*. *micrantha* during range expansion in South China.

*Mikania micrantha* increased intra‐ vs. interspecific competitive ability and intraspecific competition tolerance in response to the shifting competition environment from heterospecific to conspecific dominant, suggesting *M*. *micrantha* has a great potential for rapid adaptation to local competition environment. This result emphasizes the importance of maintaining intraspecific competitive ability on population persistence during invasion course. In fact, intraspecific competition tolerance is a promising competitive strategy for invasive plants, especially in the late invasion phase (Novoplansky, 2010), and this strategy has been reported in some invasive plants (Golivets & Wallin, 2018; Huang et al., 2018). Congruent with our results, a previous study on *M*. *micrantha* found that compared with newly invaded populations, long‐established populations produced seeds of larger mass in response to greater intraspecific competition (Huang et al., 2015). As seed mass is tightly related to seedling competitive advantage, larger seeds may benefit its growth and intraspecific competitive ability in early growth stage. In addition to the competition environment, factors that covary with the competition environment in the field, such as specialist enemy loads (Lankau & Strauss, 2011) and environmental gradients (Colautti et al., 2009), may jointly lead to shifts in competitive ability.

The negative soil biota effect was detected for *M*. *micrantha* (Figure S2), indicating that enemy release, if any, is transient during its invasion. Importantly, the results indicated that the soil biota effect on intra‐ and interspecific competitive ability was highly depending on the population's competition environment. Specifically, intraspecific competition tolerance was much less susceptible to soil biota relative to interspecific competition tolerance, especially when the competition environment shifted to conspecific‐dominant (Figure 4). Combining the result of increasing tolerance of the negative soil biota effect, one plausible explanation for this pattern is that *M*. *micrantha* may incessantly fine‐tune its interactions with soil biota, for instance, strengthened tolerance of the negative soil biota effect, as indicated by our results, to maintain intraspecific competitive ability but no longer interspecific competitive ability. A parallel experiment showed that *M*. *micrantha* reduced its responsiveness to arbuscular mycorrhizal fungal (AMF) with increasing conspecific abundance (Huang et al., unpublished data). Evidence was also found in previous studies that reduction in AMF associations promotes competitive tolerance in invasive species (Seifert et al., 2009; Shelby et al., 2016). In the case of *M*. *micrantha*, as the altered interactions with soil biota were indicated to come at costs (i.e., biomass of populations from high conspecific and low heterospecific abundance sites was less increased by the removal of soil biota than populations from the other ends, Figure 3), evolutionary processes involving enhancing resistance/tolerance of antagonistic microbial components, rather than reducing AMF dependence, may be more related to the changes in competitive ability.

Contrary to the tight interactions with soil biota in populations from sites of intense intraspecific competition, relatively weak interactions with soil biota was found in populations from sites of strong interspecific competition, despite negative soil biota effect. This difference in soil biota interactions indicates contrasting selection regimes between intra‐ vs. interspecific dominant competition environments. It is possible that in areas with mostly heterospecific competitors, the low selective pressures from competitors and soil biota release *M*. *micrantha* from evolutionary constraints and, therefore, promote *M*. *micrantha* to grow fast and achieve high biomass facilitating its establishment and spread; but in areas with mostly conspecific competitors, *M*. *micrantha* tends to tolerate high competitive conditions and negative soil biota effect, but with low growth potential in optimal conditions (MacDougall & Turkington, 2004). Consistently, our results showed that populations from low conspecific and high heterospecific abundance sites gained an obvious increase in biomass from the removal of soil biota (i.e., characteristic of fast growth), but populations from the other ends benefited little from it (i.e., costs of tolerance; Figure 3).

It should be noted that the same soil inocula and competing plants were used in this study, thus the coevolutionary potential between invasive species and local communities, as suggested in previous studies (Germain et al., 2020; Oduor, 2013), was not considered. For example, the growth of *M*. *micrantha* populations from sites of high heterospecific but low conspecific abundance may be less inhibited or benefit more if soil inocula from uninvaded areas were used; *M*. *micrantha* may have a lower growth performance if competing plants were collected from long and heavily invaded areas. Therefore, although the direction of shifts in intra‐ and interspecific competitive ability could be well predicted by our results, caution will be required when referring to the magnitude of their shifts.

Our results indicate that despite experiencing the negative soil biota effect, invasive plants may manage to persistently maintain high competition performance in local competition environments through altering its interactions with soil biota. This can provide important implications for our understanding of managing invasive species with mitigating impacts on local communities. In the case of *M*. *micrantha*, introduction of competition‐tolerant native plants that align with conservation priorities may be effective where invasive populations are long‐established and inferior in interspecific competitive ability relative to intraspecific competitive ability, whereas eradication may be effective where populations are newly invaded and fast‐growing (Lankau et al., 2009).

In conclusion, we report a rare example of shift in competitive ability mediated by soil biota during plant invasion, highlighting the importance of soil biota effect on the evolution of competitive ability. To better understand the future of plant invasion, further studies are required on the topic: for example, identifying the microbial components that influence the competitive ability of invasive species, comparing the effects of soil biota from sites varied in their invasion histories and competition conditions, and disentangling the effects of other biotic and abiotic factors that covary with the competition environment.

## CONFLICT OF INTEREST

The authors declare no conflict of interest.

## AUTHOR CONTRIBUTIONS

**Fangfang Huang:** Conceptualization (equal); Formal analysis (lead); Funding acquisition (equal); Investigation (lead); Writing‐original draft (lead); Writing‐review & editing (lead). **Qiaoqiao Huang:** Conceptualization (equal); Formal analysis (supporting); Funding acquisition (equal); Writing‐review & editing (supporting). **Xianhua Gan:** Investigation (supporting); Writing‐review & editing (supporting). **Weiqiang Zhang:** Investigation (supporting); Writing‐review & editing (supporting). **Yuedong Guo:** Investigation (supporting); Writing‐review & editing (supporting). **Yuhui Huang:** Investigation (supporting); Writing‐review & editing (supporting).

## Supporting information

Appendix S1

## Data Availability

Supporting data are available from the Dryad Digital Repository: https://doi.org/10.5061/dryad.4xgxd25b3.
