# Supplementary material for: Shift in competitive ability mediated by soil biota in an invasive plant
Source: Ecol Evol. 2021 Nov 2;11(23):16693–703. doi: 10.1002/ece3.8287 (PMC8668795; doi:10.1002/ece3.8287)
Supplement: Supplementary file 1 — Appendix S1 [file ECE3-11-16693-s001.docx]

**Appendix**

**TABLE S1** Information of selected *Mikania micrantha* populations

| Site code | Longitude | Latitude | Altitude/m a.s.l | % cover of *M. micrantha* | % cover of non-*M. micrantha* plants | Habitat |
| --- | --- | --- | --- | --- | --- | --- |
| HK | 114°7′20″ | 22°22′57″ | 145 | 78 | 63 | hillside |
| SZ | 114°3′19″ | 22°33′6″ | 8 | 31 | 68 | roadside |
| DG | 114°5′37″ | 22°54′23″ | 50 | 42 | 86 | hillside |
| GZ | 113°22′51″ | 23°3′40″ | 9 | 55 | 77 | roadside |
| KP | 112°39′10″ | 22°21′55″ | 15 | 50 | 80 | roadside |
| YJ | 111°59′32″ | 21°52′31″ | 41 | 38 | 42 | roadside |
| MM | 111°11′45″ | 21°32′15″ | 79 | 77 | 19 | wasteland |
| GZ-intra* | 113°22′28″ | 23°11′55″ | 49 | 45 | 68 | hillside |

* Individuals from this population were used as competitor in the intraspecific competition treatment

**TABLE S2** Information of soil collecting locations

| Source code | Longitude | Latitude | Average altitude  /m a.s.l | Estimated invasion time* |
| --- | --- | --- | --- | --- |
| SZS | 114°3′ | 22°33′ | 35 | ＞30 a |
| GZS | 113°22′ | 23°11′ | 50 | ＞20 a |
| KPS | 112°33′ | 22°17′ | 9 | ＜10 a |

* The estimation of invasion history was based on Wang et al. (2003) and Zhang et al. (2004)

**TABLE S3** Information of soil chemicals

| Source code | Soil treatment | pH | Organic matter/g·kg^-1^ | NH_4_-N/mg·kg^-1^ | NO_3_-N/mg·kg^-1^ | P/mg·kg^-1^ | K/mg·kg^-1^ | Ca/mg·kg^-1^ | Mg/mg·kg^-1^ | Zn/mg·kg^-1^ | Mn/mg·kg^-1^ |
| --- | --- | --- | --- | --- | --- | --- | --- | --- | --- | --- | --- |
| SZS | Live | 5.17±0.045 | 55.198±9.564 | 5.917±1.849 | 16.24±5.667 | 5.288±1.408 | 153.927±26.291 | 982.55±282.386 | 64.423±9.358 | 8.736±3.272 | 16.605±6.393 |
|  | Sterilized | 5.68±0.336 | 54.538±10.378 | 54.159±10.192 | 8.724±2.754 | 8.55±2.108 | 139.897±23.96 | 1074.025±316.861 | 61.29±7.019 | 2.686±0.732 | 63.484±10.038 |
| GZS | Live | 5.298±0.045 | 19.653±1.691 | 5.359±1.569 | 3.064±0.886 | 5.25±0.387 | 72.637±10.505 | 180.719±34.881 | 23.456±1.045 | 2.523±0.248 | 11.45±3.214 |
|  | Sterilized | 5.443±0.137 | 20.073±1.407 | 36.449±2.563 | 0.471±0.108 | 10.875±0.542 | 75.613±8.85 | 266.694±42.771 | 26.753±1.348 | 1.377±0.146 | 68.693±24.333 |
| KPS | Live | 5.233±0.041 | 27.784±1.544 | 7.525±1.552 | 1.61±0.925 | 2.575±0.564 | 62.365±11.128 | 423.241±46.887 | 30.723±4.167 | 4.148±0.681 | 11.688±1.398 |
|  | Sterilized | 4.975±0.038 | 30.54±1.789 | 49.355±1.534 | 0.379±0.006 | 7.663±1.205 | 67.242±12.755 | 547.866±31.434 | 38.135±7.915 | 2.572±0.279 | 39.575±6.349 |

**TABLE S4** Linear mixed effect model results of *M. micrantha* biomass including the effects of geographic factors

| Effect | DF | F | p |
| --- | --- | --- | --- |
| *M. micrantha* % cover (conspecific cover) | 1 | 2.031 | 0.285 |
| Competition treatment (competition) | 2 | 302.075 | <0.001 |
| Soil treatment (soil) | 1 | 65.878 | <0.001 |
| Conspecific cover × competition | 2 | 12.336 | <0.001 |
| Conspecific cover × soil | 1 | 0.224 | 0.636 |
| Competition × soil | 2 | 5.607 | 0.004 |
| Conspecific cover × competition × soil | 2 | 1.759 | 0.173 |
| Initial size of focal *M. micranth*a plant | 1 | 10.045 | 0.002 |
| Soil source | 2 | 2.467 | 0.086 |
| Soil PC1 | 1 | 0.255 | 0.614 |
| Longitude | 1 | 2.289 | 0.267 |
| Latitude | 1 | 4.083 | 0.181 |
| Altitude | 1 | 0.605 | 0.509 |
|  |  |  |  |
| Non-*M. micrantha* plant % cover (heterospecific cover) | 1 | 0.083 | 0.802 |
| Competition treatment (competition) | 2 | 300.147 | <0.001 |
| Soil treatment (soil) | 1 | 64.642 | <0.001 |
| Heterospecific cover × competition | 2 | 2.839 | 0.059 |
| Heterospecific cover × soil | 1 | 5.213 | 0.023 |
| Competition × soil | 2 | 5.477 | 0.004 |
| Heterospecific cover × competition × soil | 2 | 6.611 | 0.001 |
| Initial size of focal *M. micranth*a plant | 1 | 9.996 | 0.002 |
| Soil source | 2 | 2.333 | 0.098 |
| Soil PC1 | 1 | 0.229 | 0.632 |
| Longitude | 1 | 0.220 | 0.689 |
| Latitude | 1 | 0.384 | 0.603 |
| Altitude | 1 | 0.436 | 0.580 |

**TABLE S5** Linear mixed effect model results of *M. micrantha* relative interaction intensity including the effects of geographic factors

| Effects | DF | F | p |
| --- | --- | --- | --- |
| *M. micrantha* % cover (conspecific cover) | 1 | 9.721 | 0.148 |
| Competition treatment (competition) | 1 | 7.249 | 0.007 |
| Soil treatment (soil) | 1 | 21.468 | <0.001 |
| Conspecific cover × competition | 1 | 0.250 | 0.618 |
| Conspecific cover × soil | 1 | 2.793 | 0.096 |
| Competition × soil | 1 | 3.311 | 0.070 |
| Conspecific cover × competition × soil | 1 | 4.570 | 0.033 |
| Soil source | 2 | 18.347 | <0.001 |
| Soil PC1 | 1 | 32.466 | <0.001 |
| Initial size of focal M. micrantha in the absence of competition | 1 | 7.328 | 0.011 |
| Initial size of focal M. micrantha in the presence of competition | 1 | 4.500 | 0.039 |
| Longitude | 1 | 0.913 | 0.494 |
| Latitude | 1 | 0.735 | 0.538 |
| Altitude | 1 | 1.105 | 0.430 |
|  |  |  |  |
| Non-*M. micrantha* plant % cover (heterospecific cover) | 1 | 0.105 | 0.780 |
| Competition treatment (competition) | 1 | 7.720 | 0.006 |
| Soil treatment (soil) | 1 | 20.672 | <0.001 |
| Heterospecific cover × competition | 1 | 2.425 | 0.120 |
| Heterospecific cover × soil | 1 | 11.957 | <0.001 |
| Competition × soil | 1 | 3.463 | 0.064 |
| Heterospecific cover × competition × soil | 1 | 4.788 | 0.029 |
| Soil source | 2 | 18.887 | <0.001 |
| Soil PC1 | 1 | 31.332 | <0.001 |
| Initial size of focal M. micrantha in the absence of competition | 1 | 4.382 | 0.037 |
| Initial size of focal M. micrantha in the presence of competition | 1 | 7.311 | 0.007 |
| Longitude | 1 | 0.344 | 0.624 |
| Latitude | 1 | 0.046 | 0.853 |
| Altitude | 1 | 0.723 | 0.490 |

**TABLE S6** Linear mixed effect model results of *M. micrantha* relative competitive ability including the effects of geographic factors

| Effect | DF | F | p |
| --- | --- | --- | --- |
| *M. micrantha* % cover (conspecific cover) | 1 | <0.001 | 0.991 |
| Soil treatment (soil) | 1 | 11.592 | <0.001 |
| Conspecific cover × soil | 1 | 6.670 | 0.011 |
| Initial size of *M. micrantha* plant in interspecific competition | 1 | 0.564 | 0.454 |
| Initial size of *M. micrantha* plant in intraspecific competition | 1 | 2.917 | 0.090 |
| Soil source | 2 | 1.778 | 0.172 |
| Soil PC1 | 1 | 3.782 | 0.053 |
| Longitude | 1 | 0.178 | 0.713 |
| Latitude | 1 | 0.001 | 0.978 |
| Altitude | 1 | 0.002 | 0.970 |
|  |  |  |  |
| Non-*M. micrantha* plant % cover (heterospecific cover) | 1 | 13.225 | 0.101 |
| Soil treatment (soil) | 1 | 10.416 | 0.002 |
| Heterospecific cover × soil | 1 | 9.194 | 0.003 |
| Initial size of *M. micrantha* plant in interspecific competition | 1 | 1.862 | 0.186 |
| Initial size of *M. micrantha* plant in intraspecific competition | 1 | 2.050 | 0.156 |
| Soil source | 2 | 1.564 | 0.213 |
| Soil PC1 | 1 | 3.128 | 0.079 |
| Longitude | 1 | 3.655 | 0.236 |
| Latitude | 1 | 4.606 | 0.200 |
| Altitude | 1 | 0.212 | 0.698 |

**
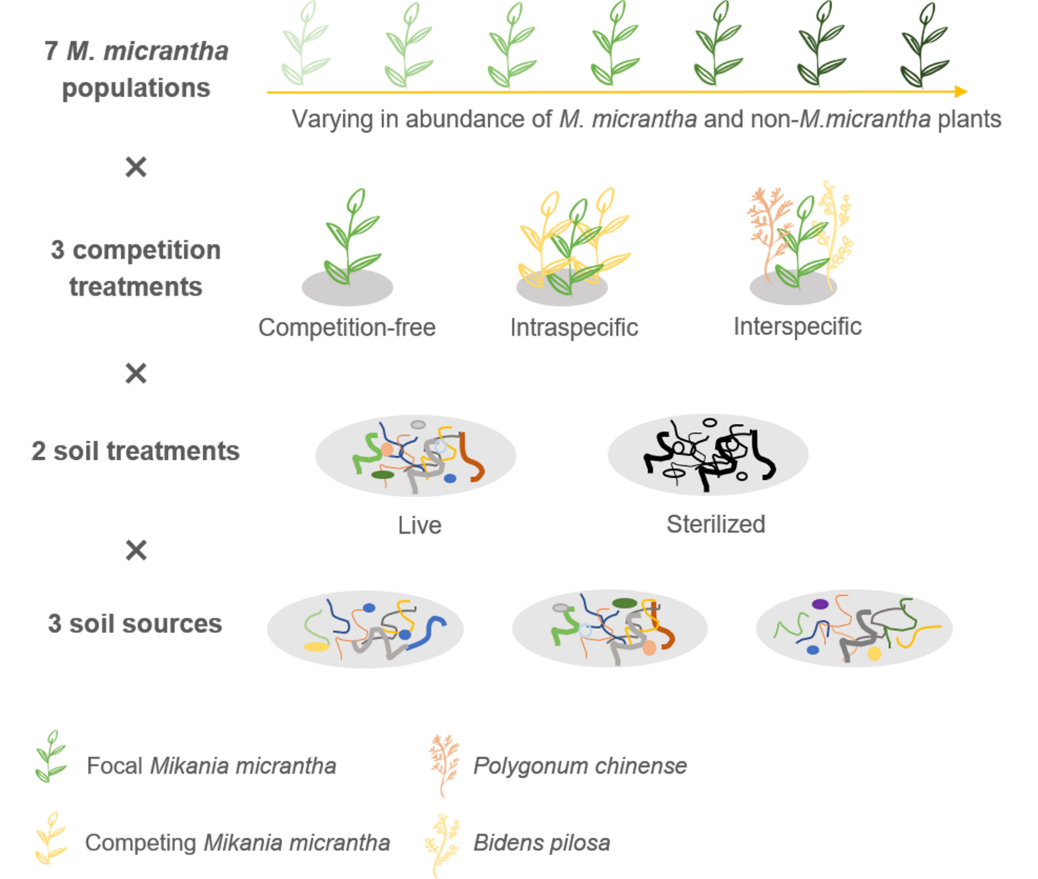
**

**FIGURE S1** Diagram of experimental design. Seven *M. micrantha* populations from different conspecific/heterospecific abundance were used. Individuals from these seven populations were grown in combinations of three competition treatments (competition-free, intra- and interspecific competitions) and two soil treatments (live and sterilized). In intraspecific competition, each plant from the seven focal populations was grown with two competing *M. micrantha* plants. In interspecific competition, each focal plant was grown with a *B. pilosa* and a *P. chinense* plant. In the soil treatment, three soil sources were used in order to reduce potential soil bias. Five replicates were set for each of the treatment combinations, yielding 630 pots (7 populations × 3 competition treatment × 2 soil treatments × 3 soil sources × 5 replicates).


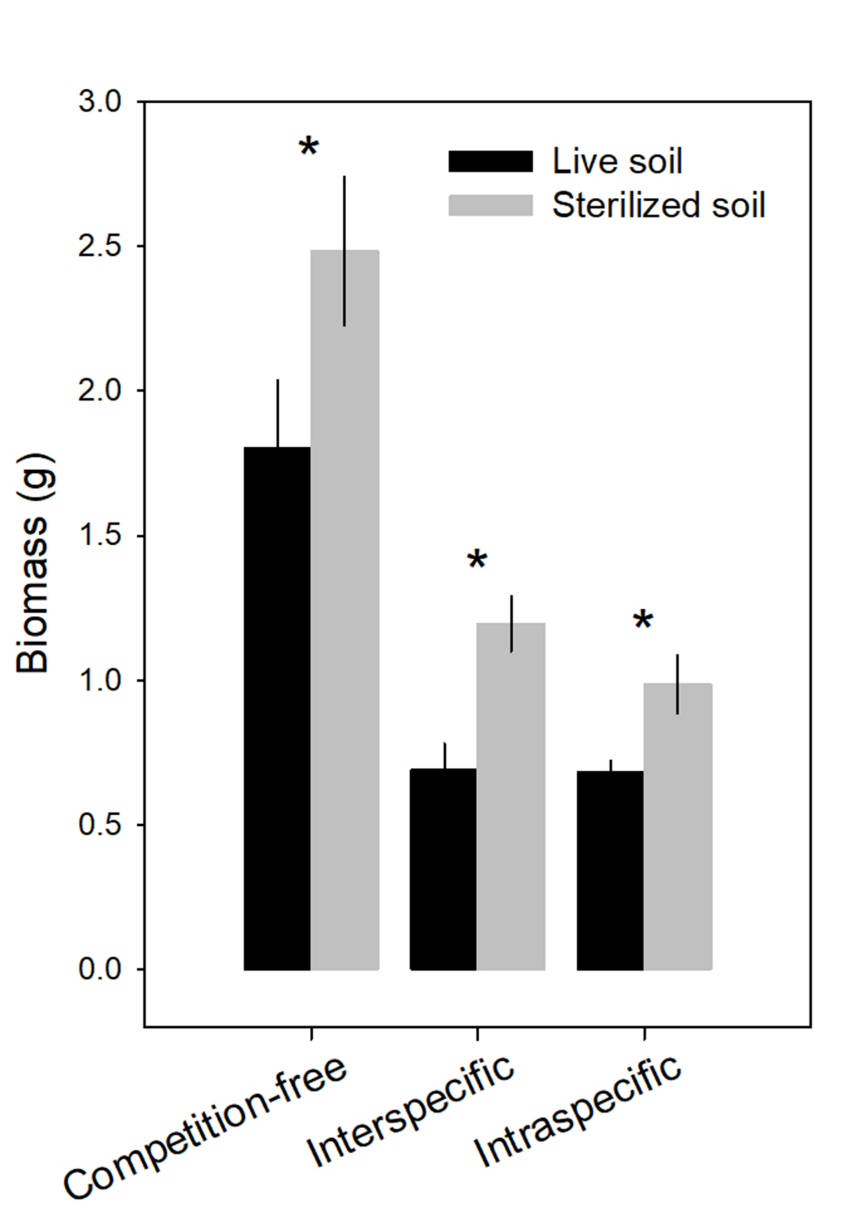


**FIGURE S2** The soil biota effect on focal *M. micrantha* biomass (mean ± SE) in different competition environments. * indicates significant difference in biomass between live and sterilized soil.
